# Supplementary material for: New admissions and asymptomatic TB cases seem to fuel TB epidemic in prisons, a cross sectional survey in Tanzania
Source: PLOS Glob Public Health. 2024 Oct 14;4(10):e0002372. doi: 10.1371/journal.pgph.0002372 (PMC11472922; doi:10.1371/journal.pgph.0002372)
Supplement: Attachment — Submitted filename: Response to Reviewers Comments.docx [file pgph.0002372.s001.docx]

PGPH-D-23-01618
New Admissions and Asymptomatic TB Cases fuel TB Epidemic in Prisons, a Cross Sectional Survey in Tanzania
PLOS Global Public Health

Dear Editor,

Thank you for taking time to review my manuscript. All the comments were really helpful and I believe have helped to improve our manuscript. We have addressed all the reviewers’ comments and highlighted our responses in yellow.

However, kindly note that we could not work on one reviewer’s comment on a revising the analysis to perform Cox hazard analysis because any major change in analysis would require approval from the prison authority which would take us few years to complete the approval process which I think would deprive us the opportunity to publish this data. However, we consider performing the analysis and this would be presented as a separate work in the future.

Below are our responses.

With kind regards

Dr. Chacha D. Mangu

Author.

==============================
EDITOR:

1. Was there a specific reason for obtain inform consent by thumb print and not signature? What was the literacy level/ education (needs to be included in table 1) of the study participants? If not all study participants were literate how was informed consent administered. What happened to participants who were diagnosed to have TB?

**RESPONSE:** In Tanzania, by law, prisoners are not allowed to sign but only using finger prints.

2. Please clarify why only 1 sputum sample (not 2 samples) was used for diagnosis. Was an attempt made for radiological diagnosis in those symptomatic but unable to produce sputum? Was any procedure used to induce sputum. The study seems to focus on pulmonary TB only, was an attempt to identify extra pulmonary TB made? The prevalence should specify 'pulmonary TB'. How was asymptomatic TB diagnosed? Kindly provide the definition of asymptomatic TB, was this also restricted to only pulmonary TB. Was an attempt to diagnose drug resistant TB made?

**RESPONSE:** When using a Xpert MTB/RIF® Assay for TB diagnosis, only one sputum sample is recommended.

3. What is the standard procedure for diagnosis and management of TB in prisons in Tanzania.

**RESPONSE:** During the time of our study, TB diagnosis was passive depending on the reporting of the symptoms and tested using smear microscope, those diagnosed positive were treated according to the national guideline. After our study, the current standard procedures included screening at the point of entry using Gene Xpert, symptomatic screening is also being done and treatment follows national guideline.

4. Was screening and diagnosis done on the day of admission to the prison or after a few days. If after a few days, were any strategies used for screening for TB in contacts within the prison-and outside as applicable.

**RESPONSE:** All prisoners entering the prison were supposed to provide sputum on the day of entry in the prison. There were some who were missed on the day of entry e.g., those who were admitted during the weekends, or when there was a backlog of samples to be screened and they could not be screened on the same day they entered the prison. Those were screening immediately when the next opportunity was available.

5. Kindly add a section on the limitations of your study (primarily methodological issues) at the end of the discussion.

**RESPONSE:** Section added
==============================

Journal Requirements:

1. We have noticed that you have uploaded Supporting Information files, but you have not included a list of legends. Please add a full list of legends for your Supporting Information files after the references list.

Response: LEGENDS added

**OUR RESPONSES TO REVIEWERS**

Reviewer #1: This is a well-written manuscript on a subject of very high interest in the field of tuberculosis. Local authorities and policymakers will benefit from this publication, and the WHO End TB strategies will benefit from this knowledge.

Some remarks:

The authors should provide more data from their country or region on the proportion of TB cases in prisons compared to the total number of cases in the country. Furthermore, it might be interesting to compare the number of TB cases that existed before in those five prisons, and how much was collected in a year through active case-finding. One way to measure the impact of this intervention and estimate the costs of implementing it periodically is to use the following method.
The title "New Admissions and Asymptomatic TB Cases fuel TB Epidemic in Prisons, a 2 Cross Sectional Survey in Tanzania" is questionable. I would suggest being less definitive, and using the phrase "seems to fuel" instead of "fuel".

Response: TB Data from the prisons in Tanzania is lacking. The baseline data were collected from the DHSI2 but cannot be used in this manuscript due to the permission issues.

Line 62, Please review current literature such: Martinez L et al “Global, regional, and national estimates of tuberculosis incidence and case detection among incarcerated individuals from 2000 to 2019: a systematic analysis.”

Response: Noted and cited

Line 90. Screening was performed upon entry or transfer from the prison. How this was done can be more detailed. This is because it is not clear what is done on the day of entry or up to six months after entering prison. It would be good to know how much time, on average, it took to perform the analysis on a new admission.

Response: Elaborated

Line 96. The questionnaire collected information on the date of admission to prison and included the date of confirmation of TB. Therefore, we have the necessary tools to perform Cox regression. Why did the authors decide to perform logistic regression?

Response: The purpose of the paper was to look at the factors associated with TB in prison settings and estimate TB burden in prison not time to a positive TB diagnosis. However, this is a good proposition and can be performed in the future and presented as a separate paper.

Line 167. These lines suggest that Ukonga prison does not receive new admissions, and Segerea does not receive convicted criminals. This also suggests that among the five prisons, the dynamics of admission and mobility are very different. Therefore, specific analyses of the variables for each prison are required. Can the authors comment on this?

Response: Both prisons Ukonga and Segerea receives new admissions but in different proportions. Prisoners sentenced for long term were sent to Ukonga and those for short term were sentence to Segerea. The sentences in the manuscript could be misleading and have been removed. The dynamics of these prisons are very much similar.

Table 2 contains the same data as in Table 1, with only a difference in how the percentage is displayed. My suggestion is to eliminate Table 1 and change it to a flowchart that explains the steps of the study about the people who took questionnaires, how many refused to participate, how many took the Xpert, then which ones were valid, and finally how many cases had TB.

Response: Table 1 contains the characteristics and TB diagnoses while table 2 shows the final outcome of the multivariable analysis. Therefore, if we remove table 1 important information on the proportion of TB cases as reflected in each variable will be missing. The idea to include a flow chart is good and I have made one.

Table 2. In the methodology, the authors state that the final model only includes variables with a p-value <0.1. But if there is the variable “number of cellmates” (p value:0.195). If all variables are going to be included, consider also important to include the “New admission” variable in Table 2. This is mainly because, owing to the chosen title, it is an important variable.

Response: The reason why we force in the number of cell mate it is because was wanted to see if overcrowding would increase the risk for TB in our model. We did not include “New admission” because the effect had already been demonstrated for this variable and hence, we therefore concluded that it is rather better to assessed new admission through the duration of incarceration (<6months).

Line 186. TB symptoms were the best predictors of TB (4.07 vs 1.06). However, 57% (122/214) of the diagnosed patients had no symptoms. This is perhaps the most important aspect of the present study. Therefore, the author must discuss it more and perhaps review some limitations in the collection of symptoms, knowledge, language barriers, and so on.

Response: This aspect was followed much later after the study where we appreciated the role of opioids in suppression of TB symptoms. Since we did not collect information on the use of opioids, we could not verify whether this was an effect in our study. However, the possibility is huge due to the fact that most of the offenders come from the people who use drugs. We have highlighted this fact and added a sentence and more citations.

Line 188. The authors say this: “Therefore, an effective screening intervention is one that applies mixed methods that detect both symptomatic and asymptomatic tuberculosis.” Have you discussed how often it should be done, the approximate cost, and what would you recommend for LIC and LMIC countries? There is literature on this, mainly from Brazil. This is discussed in the conclusion section.

Response: Mixed methods referred to here is using both symptomatic and sputum screening. Cost effectiveness study was beyond the scope of this paper. It is obvious that, using Xpert for TB entry screening is expensive, the use of the screening methods to select cases that will require Xpert is necessary. Such modalities such as X-ray can be investigated to understand how effective they are. This is why the statement in the conclusion is left open so that such modalities can be considered depending on the resources available in the country. We have made a conclusion that further studies are need to assess the impact and cost effectiveness of using WHO approved rapid diagnostics for TB screening in prison settings.

Line 190- To review. There were 9077 new admissions, and 132 (1.45%) had TB. Prisoners under 6 months of age comprised 62.3% (8643/13763) of the total population. This group comprised 53.3% (114/214) of the TB cases. New admissions have a lower prevalence of TB than previous prisoners. In absolute numbers, there are more because 66% of the population came from a new admission. However, in terms of rates, it was lower. The risk of developing tuberculosis increases over time in prisons. The TB notification rate of the group of those who spent less time was lower when compared to those who spent more time in prison and below the global rate found (1555/100 thousand inhabitants). Therefore, I conclude that the incidence rate is very similar in all groups, but increases with time in prison and with the number of readmissions.

It is a significant aspect to note that the prisoner turnover is quite high. Approximately 2 times its static size. The median time spent in prison was 1.5 months. This means that a large part of the population is always new, and everything that occurs in it will be the majority. Nevertheless, in terms of relative comparison, it does not hold true.

It is important and appropriate for authors to address certain limitations of the study.

Response:

Reviewer #2: Dear Author,
Thank you for sending the manuscript. These are some of the observations and suggestions made based on my knowledge and understanding of the paper. Please check and do the needful.

Abstract
28: No mention of early morning samples in the main text

Response: The WHO recommends only one spot sample whenever using Gene Xpert. This was the reason why there were no early sputum samples collected.

36: Error in the calculated prevalence, please correct

Response: 214/13,763 = 1.55489356

Introduction
52-54 : Please add reference

Response: This is the authors’ statement hence using “most likely”

55: Updated statistics available, please add the latest numbers

Response: Updated

72-75: Split the long sentence into two for better understanding and mention if it is universal screening for all new inmates irrespective of whether they are symptomatic or not?
* All references needs to be in square brackets according to the author guidelines of journal

Response: Ok, done

Methods
90-92: Please define new admission to prison and on an average how many days after enrolment were they screened if that data is available

Response: We are sorry, data is not available

94: Is the screening tool used a validated questionnaire?

Response: The screening tool used was a standard WHO 5 symptoms questionnaire. For study specific purpose, questions were added to collect additional data used in this analysis. However, this was used to collect information on the diseases presentation and not screening to where she/he will be allowed to take part in the study.

95: Please remove full stop in between the sentence

Response: Sentence rephrased, thank you.

99: Please explain why two sputum samples were not collected for all according to the diagnostic guidelines

Response: According to WHO guideline for use of Gene Xpert, only one spot sample is adequate. (WHO operational handbook on tuberculosis. Module 3: Diagnosis - Rapid diagnostics for tuberculosis detection 2021 update)
*There is no mention of the minimum required sample size for the study. Please include sample size calculation also and the reference for the same

Response: This was an intervention study implementing screening procedure. Therefore, there was no sample size but all the eligible participants were offered to receive TB screening. This was due to two main reasons, first, it was considered unethical to select few prisoners to receive an intervention that was considered beneficial to all prisoners. And secondly, to achieve infection prevention and control, it is empirical that all TB cases are identified and treated, to select a sample size would have posed a possibility of leaving some TB cases undiagnosed.

Ethical consideration
*Better to be mentioned before the study procedures

Response:

Results
141: It would be more explanatory if the total number of prisoners approached, number consented and number screened is added to know the missed numbers as well.
142: out of 16312 consented, why only 13868 were tested? What about the remaining 2264?

Response: A flow chart has been created.

154:
Table1
Number of cellmates: total numbers have an error
Prison ID: Please check the prevalence rates, disparity noted.

Response: Corrected, Thank you.

174:
Table 2
Number of incarcerations :spelling mistake

Response: Corrected, thank you.

Discussion:
177: "more than" "higher than" both phrases mentioned in the same sentence
180-183:Split the sentence for better understanding

Response: This has been done, thank you.

Thank you

Reviewer #3: Authors are presenting important findings from a multi-site active TB case finding survey in Tanzanian correctional system. Below are recommendations to make the manuscript suitable for publication:

OVERALL
=====
- The manuscript would benefit from a thorough proof-reading
- Authors are advised to use a humanising language for people in incarceration. We are trying to avoid "inmates" or "prisoners" and replace them with "incarcerated individuals" or "people who are incarcerated". Please have a look though this article. Tran, N.T., Baggio, S., Dawson, A. et al. Words matter: a call for humanizing and respectful language to describe people who experience incarceration. BMC Int Health Hum Rights 18, 41 (2018). https://doi.org/10.1186/s12914-018-0180-4

ABSTRACT
=======
- Lacks a sentence describing the aims of the study
- Provide TB case definition

INTRODUCTION
==========
- Proper review of relevant literature is required
- Provide local (Tanzanian) TB data, including available TB in prison information

Response: Thank you, this has been done for data that is available. Local prison data are not avialalble.

METHODS
======
- Describe how the already incarcerated individuals were recruited? It seems that only half of this population was recruited (4000 out of 8000 individuals). This attracts a potentially major source of selection bias, if particular individuals were selected or only those presented to prisons' clinics!
- Similarly, describe why these prisons in particular were chosen? randomly? What is so special about them?

Response: All prisoners were offered an opportunity for screening. There was neither selection or randomization.

- The use of prison staff in administering the questionnaire and collecting the sputum specimens might have jeopardised the confidentiality of participants and caused a breach of your ethical statement. Can you elaborate further on this!

Response: Prison staff referred were health workers who workers in the prison health facilities of which were mainly prison staff. These staff were trained on research ethical principles including GCP to ensure that all ethical principle were adhered to during the study period. Moreover, frequent supportive supervisions were performed to ensure that the study was conducted according to the protocol.

- It is very important to describe the TB symptoms collected! What were they?

Response: Classical TB symptoms used for symptomatic screening were collected, cough, fever, weight loss, night sweats and hemoptysis.

- Describe whether the sputum handling was done in accordance with the manufacturer's guidelines.

Response: Sputum handling was performed according to SOP which was compliant to the manufacturer requirements.

- Justify the categorisation of each variable to the described categories. e.g age, times of incarceration...etc.

Responses: These were variable selected according to their relationship to TB

- It would be very good to the model to adjust findings to prisons, instead of using prisons as a variable.

Response: This was done in the multivariable analysis

- More information is required on the multivariable regression analysis. Did you use forward/backward handling of variables? How did you choose the final model?

Response: This was explained; it was a forward handling of variable and every variable with p-value of <0.1 was selected and included in the final model.

RESULTS
======
- It is not possible to consider the finding for the study as the prevalence of TB (or sometimes you use the burden of TB) in these prisons, unless you further explain the recruitment process of the already incarcerated individuals. If is not involving ALL, then you can simply say "prevalence of TB in the screened sample", which can be the closest to a representative sample.
- Can you further describe the meaning to the "duration of incarceration"? Is the sentence duration or the period spent already in prison.

Response: Duration of incarceration is the time spent in prison.

DISCUSSION
========
- No section describing the limitations of the study!
- Limited description of the findings.
- Needs further review of the literature to add relevant details.
- The use of symptoms to define TB cases in prisons might have poor utility and might underestimate the true prevalence of Tb (See PMID: 27197601). In your study more than half (N=112) of TB cases would be missed if relied on TB symptoms.

Response: This has been done, thank you.

CONCLUSION
=========
- As above, further information is required in order to consider the finding as the prevalence of TB in the prison system.
