# Supplementary material for: New admissions and asymptomatic TB cases seem to fuel TB epidemic in prisons, a cross sectional survey in Tanzania
Source: PLOS Glob Public Health. 2024 Oct 14;4(10):e0002372. doi: 10.1371/journal.pgph.0002372 (PMC11472922; doi:10.1371/journal.pgph.0002372)
Supplement: Attachment — Submitted filename: Response to Reviewers.docx [file pgph.0002372.s002.docx]

Chacha David Mangu

P.O. Box 2410,

Mbeya.

17^th^ March 2024

Editor, PLOS Global Public Health,

Dear,

RE: PGPH-D-23-01618R1, Revisions for New Admissions and Asymptomatic TB Cases Seem to fuel TB Epidemic in Prisons, a Cross Sectional Survey in Tanzania


Dear Editor,

Thank you for taking time to review our manuscript. Your inputs are always very helpful and similarly this second round of comments helped to improve our manuscript. We have addressed all the reviewers’ comments and highlighted our responses in yellow.

We have re-clarified the comments that were raised in the previous review and improved the sections that we think needed more clarification in the manuscript. I hope this will make the manuscript clearer.

Below are our responses highlighted yellow.

With kind regards

Dr. Chacha D. Mangu

Author.

**RESPONSES**

6. Review Comments to the Author

Please use the space provided to explain your answers to the questions above. You may also include additional comments for the author, including concerns about dual publication, research ethics, or publication ethics. (Please upload your review as an attachment if it exceeds 20,000 characters)

Reviewer #2: Thank you for replying to the comments and making the suggested edits.
These are some observations on the comments:

94- Standard screening tools are generally used for screening for a particular disease/ condition.It has sensitivity and specificity. If you have made a questionnaire with questions prepared by yourselves, it would be great if you pilot test and face validate the questionnaire with the help of an expert.If this is followed, please mention it in the manuscript or mention it as a limitation of the study

RESPONSE: The WHO standard symptom screening questions were used. This has been updated in the manuscript.

99- It cannot be mentioned as an intervention study: screening for a disease and doing lab investigations to confirm the screen positives is not intervention.

Sample size calculation: The ultimate goal of achieving optimum sensitivity in terms of power and confidence in an experiment requires consideration of the optimum number of subjects included(i.e. minimum sample size.

RESPONSE: In this study, we were implementing a screening protocol using a combination of screening questionnaire and Xpert for all the inmates in central prison. Our funding requirement were to screen all the prisoners incarcerated during the funding period to (i) detect TB cases in prison (ii) Estimate the burden of TB in central prisons. This is why we have stated in our procedure that ALL prisoners residing during study period were offered screening opportunity and nearly all of them agreed (this is one of the limitations I have highlighted).

Sampling technique: the sampling technique that you are using in the selection of your estimated sample size is different in different scenarios based on the appropriateness. Hence this may not be a correct explanation for the same.

Hence its always better to calculate the minimum sample size.

Response: We analyses data from a study where ALL prisoners were screened. Sampling was against our funding requirements.

141- In the flow chart you have mentioned, 2264 excluded because they were not able to produce sputum. explain &quot;105 invalid or error&quot; in what?-sputum sample collection? Please incorporate the necessary details written in the text lines 153-156 to methodology flow chart so that it is self-explanatory.

RESPONSE: It has been stated, “error results”

Please include the limitations of the study considering all the factors reviewers have commented.

All the best!

Reviewer #3: I have noticed that the manuscript has improved, but most of my comments on the earlier version were answered in the reply letter, but recommendations were not incorporated in the text. Comments made by the reviewers are meant to improve the manuscript and make it suitable for publications not to be answered in a reply letter.
For example:
- Please incorporate in the text that only half of the approached incarcerated individuals consented for the screening.

RESPONSE: This is incorrect; the prisoners would house about 8000 prisoners, but when taking into account those who are admitted and being discharged (who would most be short term remandees), the prisons would have a bout of more than 15000 individuals per year. We were able to screen 16,000 incarcerated individuals. The classification we provided of inmate and new admissions was based on whether the individual was admitted before or after the screening started. It took about 6 months to finish screening all the prisoners in the respective prison.

- Please include in the limitations that the use of prison&#39;s healthcare worker might attract a confidentiality breach, but for logistic (?) reasons, they were used! Even if they were trained on ethical principles, they remain a source of confidentiality breach, given their representation of the prison department.
- Explicitly present the TB symptoms you described in the letter inside the manuscript text.

Response: This has been added in the limitation

- My question about the categorisation is not about the choice of variables, but rather the numbers used in each category cells. ? have equal numbers, ? using median... etc

Response: Categorization was decided using a combination of factors. E.g. We used more about 6 month to complete screening of the whole prison, therefore we categorized time spent in prison <6month and ≥6months to separate most of the new entries; age categorization was systematic with range of 10 years; etc.

- Change variable of duration of incarceration to &quot;time spent in prison&quot;, the first one is misleading as it might imply what they were sentenced with, which is different.

Response: “Duration of incarceration” has been changed to “Time spent in prison”.
