# Supplementary material for: New admissions and asymptomatic TB cases seem to fuel TB epidemic in prisons, a cross sectional survey in Tanzania
Source: PLOS Glob Public Health. 2024 Oct 14;4(10):e0002372. doi: 10.1371/journal.pgph.0002372 (PMC11472922; doi:10.1371/journal.pgph.0002372)
Supplement: Attachment — Submitted filename: Response to Reviewers.docx [file pgph.0002372.s003.docx]

**Dear Editor,**

RE: PGPH-D-23-01618R2
New Admissions and Asymptomatic TB Cases Seem to fuel TB Epidemic in Prisons, a Cross Sectional Survey in Tanzania (Mangu *et al.*)

**I acknowledge receiving comments from the reviewers that need to be addressed as follows:**

4. Have the authors made all data underlying the findings in their manuscript fully available (please refer to the Data Availability Statement at the start of the manuscript PDF file)?

The [PLOS Data policy](https://journals.plos.org/globalpublichealth/s/data-availability) requires authors to make all data underlying the findings described in their manuscript fully available without restriction, with rare exception. The data should be provided as part of the manuscript or its supporting information, or deposited to a public repository. For example, in addition to summary statistics, the data points behind means, medians and variance measures should be available. If there are restrictions on publicly sharing data—e.g. participant privacy or use of data from a third party—those must be specified.

Reviewer #2: No

RESPOSE: This data is from a vulnerable population which is protected by law. This data cannot be shared publicly. However, arrangement for data access can be made through the national health research commission confidential under the law. However, full address of the Ethical committee has been shared and data can be available upon request.

Reviewer #3: Yes

6. Review Comments to the Author

Please use the space provided to explain your answers to the questions above. You may also include additional comments for the author, including concerns about dual publication, research ethics, or publication ethics. (Please upload your review as an attachment if it exceeds 20,000 characters)

Reviewer #2: 41- Since the prevalence of TB was more among the inmates compared to new admissions
(as mentioned in the results) and there is no significant association found between duration of length of stay (if new admissions is defined as <6months) the statement "new admissions could be the driving force of TB epidemic" in the conclusion of abstract cannot be made. Please add the answer to your objective as the conclusion.

RESPONSE: Despite that the prevalence of TB among inmates was high among those incarcerated for more than 6month, there were more incident cases detected among the new admissions. This is a reason why we made this conclusion that, new admission could be driving the TB epidemic in prison due to the fact that the introduction of more infectious TB cases come from outside the prison.

24,62,178- Prevalence of TB in Tanzania/prisons is mentioned as prevalence per lakh population as well as in person years in the abstract, introduction and discussion. I think its better to use the same unit throughout.

RESPONSE: This has been corrected. For the summary, results and discussion sections, prevalence from study is presented per 100,000 population. In the introductions, incidence or prevalence rates have been cited as presented in papers

Reviewer #3: Authors have addressed comments raised previously.

Thank you,

With kind regards

**Dr. Chacha Mangu.**
